# Weaning patients off long-term prednisolone: a survey of physicians’ practice in the UK and Southeast Asia

**DOI:** 10.1136/bmjopen-2025-107269

**Published:** 2025-12-30

**Authors:** Katharine Lazarus, Pei Chia Eng, Kavita Narula, Angelica Sharma, Sirazum Choudhury, Deborah Papadopoulou, Niamh M Martin, Florian Wernig, Tricia Tan, Karim Meeran

**Affiliations:** 1 Department of Endocrinology, Imperial College Healthcare NHS Trust, London, UK; 2 Department of Diabetes, Endocrinology and Metabolism, Imperial College London, London, UK; 3 Department of Endocrinology, National University Hospital, Singapore

**Keywords:** Adrenal disorders, General endocrinology, Diabetes & endocrinology

## Abstract

**Abstract:**

**Study objective:**

Prolonged glucocorticoid (GC) use is associated with significant morbidity and mortality, including the development of GC induced adrenal insufficiency. Recent guidance from the European Society of Endocrinology and Endocrine Society provides a framework for tapering GCs. However, there is limited understanding of current practice across endocrine and other medical specialties, including barriers and challenges to GC weaning. This study aimed to establish how GCs are weaned in patients across endocrine and non-endocrine specialists.

**Design and setting:**

Anonymous online surveys were disseminated to all members of the Society for Endocrinology and all members of the Association of Southeast Asian Nations Federation of Endocrine Societies and the Endocrine and Metabolic Society of Singapore. Non-endocrine specialists were surveyed in the UK and in Singapore.

**Results:**

A total of 306 (258 endocrine specialists and 48 non-endocrine specialists) responded to the survey. Approaches to discontinuing prednisolone were heterogeneous. Among endocrine respondents, only 78% would fully wean the prednisolone, with 50.4% switching to hydrocortisone to wean and 12.6% favouring long-term GC replacement without further investigations. Among the non-endocrine respondents, 16.7% would stop prednisolone abruptly and 10.4% would refer to endocrinology to supervise weaning. The most common barrier to weaning GCs reported by both endocrine and non-endocrine specialists was relapse of the underlying condition (55.9% and 70.8%, respectively).

**Conclusions:**

Relapse of the underlying condition is common, and endocrinology input may not be appropriate when this occurs. There remains a need to develop an evidence-based approach for safe and effective GC weaning and hypothalamic–pituitary–adrenal axis assessment.

**STRENGTHS AND LIMITATIONS OF THIS STUDY**

- A web-based survey capturing glucocorticoid (GC) weaning practices among endocrine and non-endocrine specialists in the UK and Southeast Asia.
- Limitations include the respondent time frame of 3 months and design of the survey in English, which may have limited participation.
- The design of the survey relied on self-reported data, which may be subject to recall bias.
- The survey explores real-world barriers to GC weaning, which will inform the design of larger prospective GC weaning studies.

## Introduction

 Prolonged use of glucocorticoid (GC), even at low doses (eg, prednisolone 5 mg daily) is associated with significant adverse outcomes, including increased morbidity and mortality.^1^ These risks include an increased risk of osteoporosis,^2^ diabetes, cardiovascular disease, stroke and death.^3^ The resultant healthcare burden is substantial, contributing to increased economic costs.^4 5^ Prolonged administration of exogenous GC fuels GC-induced adrenal insufficiency (GC-AI) through suppression of the hypothalamic–pituitary–adrenal (HPA) axis. This condition represents the most common cause of adrenal insufficiency, surpassing classical aetiologies such as Addison’s disease or pituitary disorders.^6^

Recent guidance from the European Society of Endocrinology and American Endocrine Society distinguishes between a therapeutic and physiological (endocrine) GC taper and advises weaning patients to a physiological dose of GCs prior to evaluating the HPA axis.^7^ The guidance provides a choice of two scenarios to guide weaning: (1) GCs should be gradually tapered and patients monitored for clinical signs of AI or (2) patients should undergo routine biochemical testing with a morning serum cortisol. The guidance advises that if a morning cortisol value is >300 nmol/L, then GCs may be stopped safely. If a morning cortisol value is 150–300 nmol/L or <150 nmol/L, then this should be repeated in a few weeks or months, and the current GC dose continued.^7^ Endocrinology referral is recommended for those with a history of adrenal crisis or those who fail to achieve HPA axis recovery after 1 year receiving a physiological GC dose.^7^ Additionally, the guidelines recommend that adults weaning prednisolone should not be switched to replacement hydrocortisone. Despite these recommendations, there is limited understanding of their implementation in clinical practice, including the barriers to effective GC weaning and the appropriateness of endocrinology referrals.

The evidence for optimal GC withdrawal strategies remains limited. Existing studies are heterogenous, with significant variability in withdrawal durations, GC types, tapering methods and study populations. Moreover, most studies lack clear guidance on tapering protocols once patients reach physiological GC doses.^8^

To understand these gaps, this study aimed to evaluate current practice in the management of long-term GC weaning by endocrinologists and non-endocrine specialists within the UK and Southeast Asia (SEA). Specifically, we compared how patients no longer requiring long-term prednisolone would be investigated and managed and identified some of the real-world barriers to successful GC weaning.

## Materials and methods

### Study design

We conducted an anonymised voluntary survey that was electronically disseminated between May and July 2024 to endocrinologists and non-endocrinologists who manage patients undergoing a GC wean. The practising healthcare professionals were identified from professional groups (eg, Society of Endocrinology, UK, Endocrine and Metabolic Society of Singapore, Singapore, Association of Southeast Asian Nations of Endocrine Society), and continuous professional development events (eg, internal grand rounds). These regions were chosen as both have high GC use for chronic inflammatory conditions, but differing healthcare structures and access to diagnostics. Our teams also have established clinical networks in both regions to enable recruitment, and we aimed to capture variation in practice across contrasting resource settings.

The survey was created using Survey Monkey (SVMK, San Mateo, California, USA) and Form.sg (Singapore). It was electronically disseminated to healthcare professionals practising in the UK and Southeast Asia. An initial invitation email explained the rationale of the study with a link to the survey enabling voluntary participation. Participants could only complete the survey once. Survey responses were anonymously collected and stored electronically by the survey service, which was only accessible in a password-protected manner^9^ (see online supplemental materials).

### Survey questionnaire

**Figure 1:**
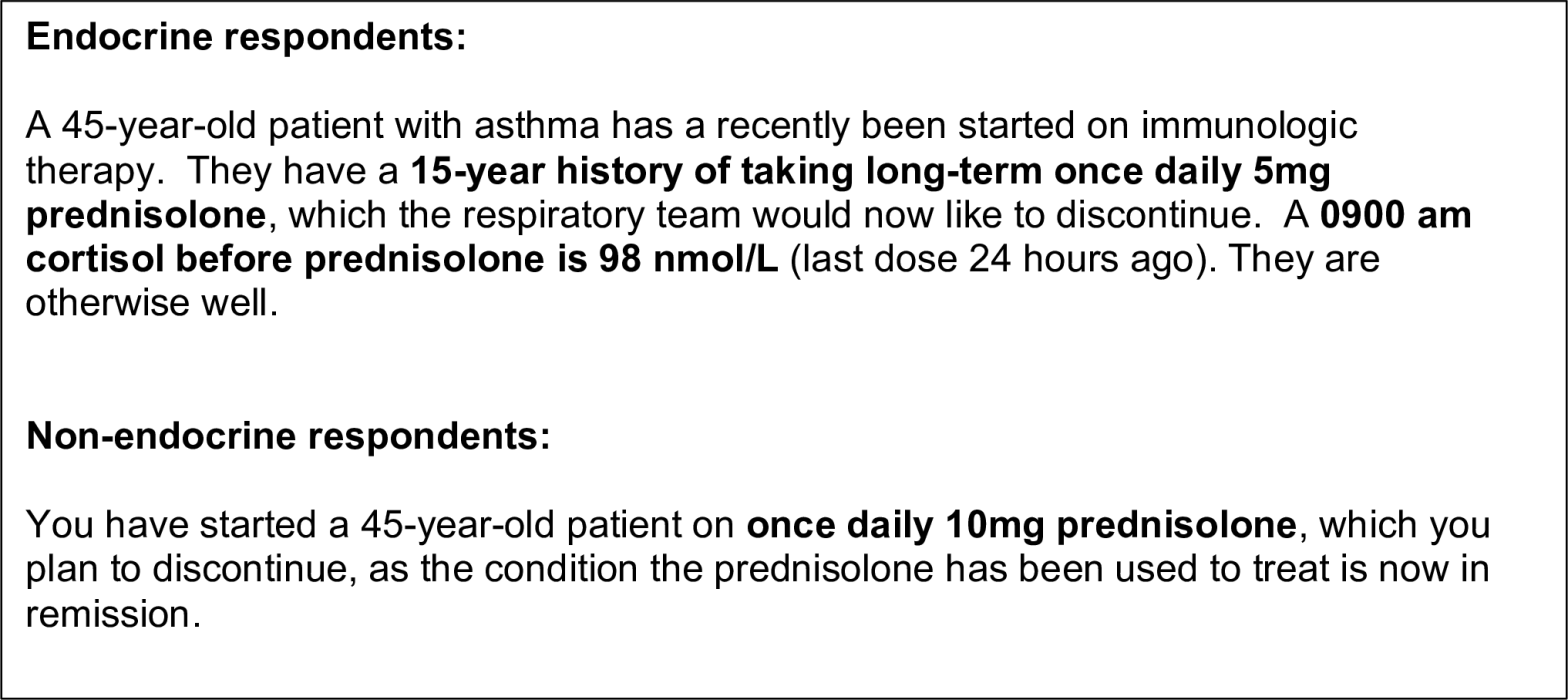
Scenarios presented to endocrine and non-endocrine respondents. Respondents were asked a series of questions around the investigations and management of patients no longer requiring prednisolone. Endocrine respondents were asked about a patient specifically taking 5 mg prednisolone with a cortisol value of 98 mmol/L while non-endocrine respondents were asked about discontinuing 10 mg prednisolone.

The survey was initially designed based on local practice and then circulated to endocrine experts to ensure it was reflective of current practice. Iterations of the revised survey were then circulated until final consensus. Given the variability in weaning protocols among different clinical specialties, we devised a common case scenario reflective of day-to-day practice to evaluate how participants would manage GC weaning. The survey questions sought to gather quantitative and qualitative data on physicians’ current practice for tapering GCs, such as prednisolone, including the investigations used to guide decision-making and to identify barriers physicians face during the weaning process. To achieve these aims, the survey included a case-based scenario of an adult patient no longer requiring prednisolone for their underlying condition, summarised in figure 1. We focused on prednisolone because it is widely used for chronic indications in many regions and, in many services, patients on longer-acting agents are routinely switched to prednisolone before tapering.

Participants were asked how they would manage GC weaning in this case. This scenario allowed the survey to capture a range of responses regarding the speed and method of tapering, the use of endocrinology referrals and additional measures such as choice of investigations during the weaning process. The full questionnaire can be found in the online supplemental materials.

### Statistical analysis

Answers to individual questions were completed on an optional basis in order to maximise participation. All returned surveys were included in the analysis. Statistical analysis was conducted using Microsoft Excel (V.16.101.3, Microsoft).

For additional analysis, survey respondents were further subcategorised into those who were endocrinologists (including clinical nurse specialists) and those who were non-endocrine specialists working across other internal medicine specialties. Descriptive statistics were used for quantitative analysis. Parameters were reported as (%, n) for categorical data and median (IQR) for continuous data. Qualitative analysis was undertaken to identify themes in the use of investigations and barriers to GC weaning.

### Patient and public involvement

The views of patients who have previously undergone GC weaning were sought at the conception of the study to better understand some of the challenges associated with weaning. Patients were not directly involved in this study as the aim was to evaluate clinicians’ approaches to weaning.

## Results

### Profiles of respondents

A total of 306 healthcare professionals responded to the survey. Of these, 258 worked within endocrinology and 48 were non-endocrine specialists treating patients requiring GCs (table 1).

**Table 1:** A description of the roles and place of work of the 258 endocrine specialists respondents in the UK and Southeast Asia (SEA) and the specialties of the 48 non-endocrine respondents

Endocrine respondents			
	UK	SEA	Total (percentage, %)
Professional role			
Consultants	130	58	188 (72.9)
Residents and trainees	24	14	38 (14.7)
Allied Healthcare Professionals	28	4	32 (12.4)
Place of practice			
Tertiary Centre	73	45	118 (41.9)
District General Hospital	89	19	118 (45.7)
Private Care	0	12	12 (4.7)
Not answered			20 (7.8)
Respondents from SEA			
Singapore		60	
Vietnam		7	
Malaysia		3	
Philippines		1	
Others (not stated)		5	

Non-endocrine respondents (UK and Singapore)	
	Total (percentage, %)
Acute medicine	10 (20.8)
Dermatology	5 (10.4)
Rheumatology	5 (10.4)
Respiratory	4 (8.3)
Gastroenterology	7 (14.6)
Haematology	2 (4.2)
Neurology	9 (18.8)
Nephrology	1 (2.1)
Not Indicated	5 (10.4)

### Investigations conducted by endocrinologists during GC weaning

**Figure 2:**
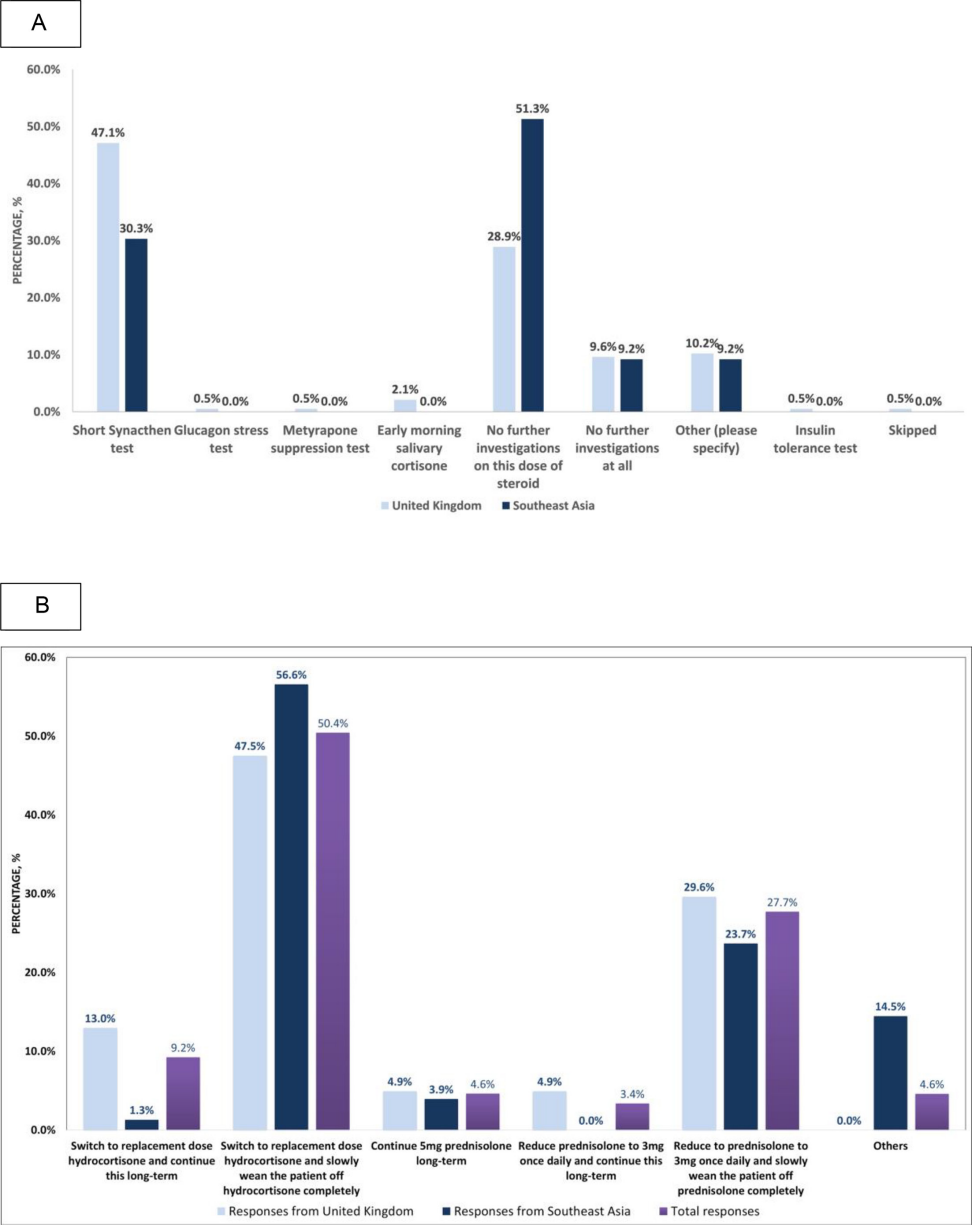
(A) Initial investigations conducted by endocrine respondents in the UK (182 respondents) and Southeast Asia (76 respondents) when assessing a patient no longer requiring long-term prednisolone 5 mg. (B) The strategies used for steroid weaning among the survey responders.

The survey sent to endocrinologists consisted of a scenario presented in figure 1. When responders were asked which investigations they would undertake in a patient no longer requiring long-term prednisolone with a morning serum cortisol level of <98 nmol/L, a Short Synacthen Test (SST) was the choice in 42.2% of respondents (figure 2A). 35.4% would wean further before assessing the HPA axis, and 9.5% would accept this morning cortisol as confirmatory of adrenal insufficiency. 9.5% would not further investigate and would continue the current dose of GC.

### Management conducted by endocrinologists during GC weaning

Figure 2B compares the proportion of respondents in the different regions, demonstrating the wide variability in GC weaning practices. The most common approach was to switch to replacement-dose hydrocortisone (50.4%) and then slowly wean off completely, compared with 27.7% who opted to wean on prednisolone. A small proportion (4.6%) said they would continue long-term 5 mg prednisolone compared with 3.4% who would reduce the prednisolone dose to 3 mg first and then continue this long-term.

### Endocrine investigations during GC weaning

Most respondents (69.2%) would repeat an SST at intervals to reassess HPA axis recovery. Only UK respondents (87/161, 36.7%) considered repeating a 09:00 cortisol measurement for HPA axis recovery, with one UK respondent considering an insulin tolerance test. Notably, 7.6% of the respondents would not carry out any investigations for HPA recovery, citing a basal cortisol of less than 100 nmol/L as sufficient to confirm adrenal insufficiency, with a need to continue long-term GC replacement.

In SEA, limited access to 1 mg prednisolone led to alternative weaning strategies, such as reducing prednisolone from 5 mg to 2.5 mg daily, then to alternate day dosing. In Vietnam, where Synacthen is unavailable, a morning cortisol value of greater than 270 nmol/L was used to guide weaning. In Singapore, some tapered to 10 mg hydrocortisone before reassessing the HPA axis.

### Investigations and management during GC weaning among non-endocrine specialists

Among non-endocrine specialists, approaches to discontinuing prednisolone were also heterogeneous. 16.7% of respondents would stop prednisolone abruptly, 47.9% said they would wean slowly (eg,1 mg per month) whereas 35.4% said they would taper more rapidly (eg,1 mg over 2 weeks). Another 10.4% of the respondents would refer to endocrinology to supervise weaning while 12.5% would check a random or early morning cortisol. 14.6% of the respondents would arrange an SST.

### Availability of GC weaning protocols

Most endocrine respondents did not have a local steroid weaning protocol. Over half said they would continue to follow up patients until the prednisolone was fully weaned. 92/234 (39.3%) would follow up 3-monthly, 108/234 (46.2%) 6-monthly and 33/234 (14.1%) would follow up at 12-monthly intervals.

### Barriers to GC weaning

**Figure 3:**
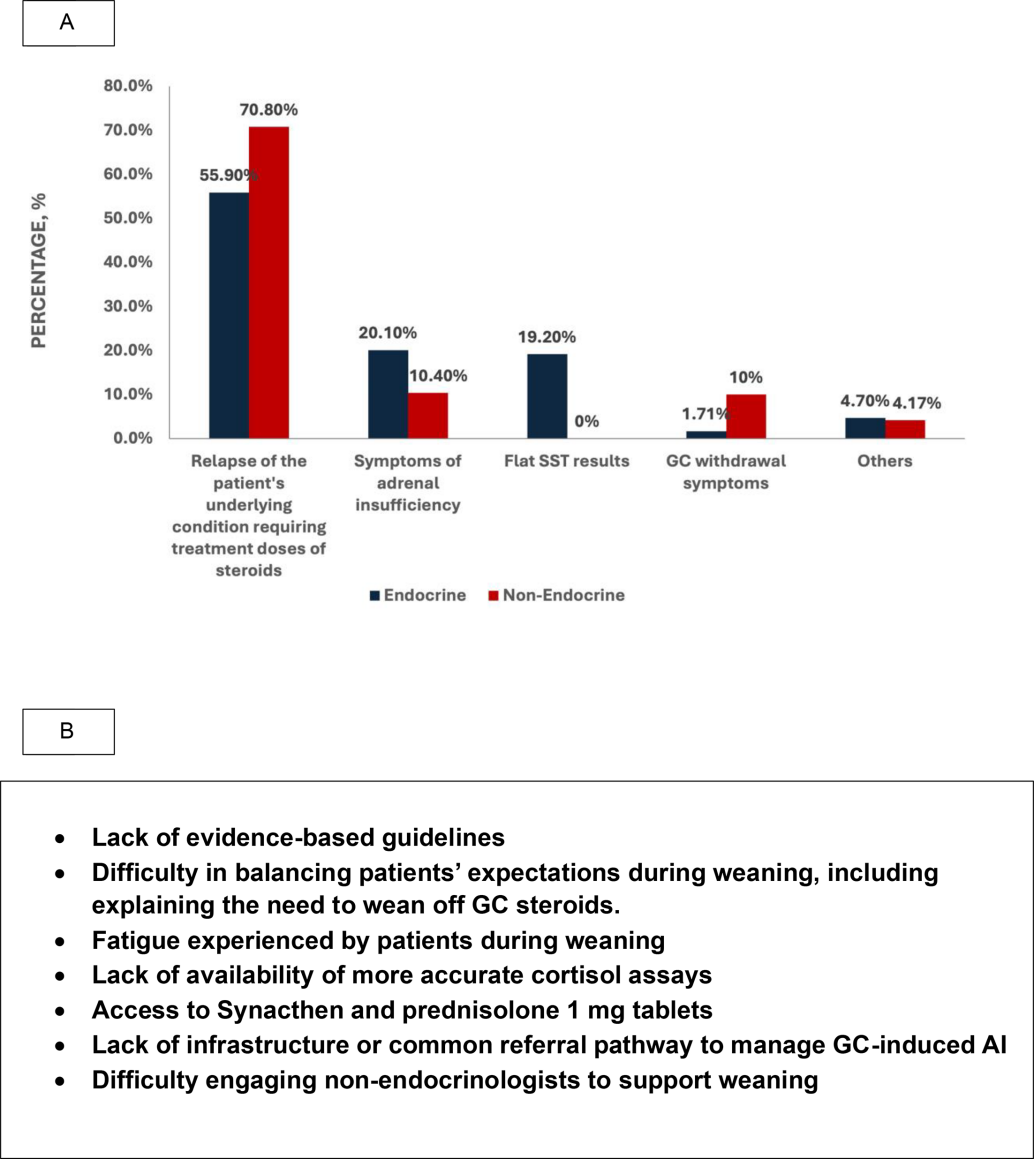
(A) The most common reported reasons for failure of glucocorticoid (GC) weaning among endocrine and non-endocrine specialists. (B) A summary of other challenges and barriers to weaning glucocorticoids highlighted by both endocrine and non-endocrine respondents.

The most frequently reported barrier to weaning was relapse of the underlying condition (55.9% of endocrine specialists and 70.8% non-endocrine specialists) (figure 3A). Symptoms of adrenal insufficiency were also commonly reported by both endocrine (20.1%) and non-endocrine specialists (10.4%), with 19.2% of endocrine respondents not attempting to wean at all if there was evidence of HPA axis suppression on dynamic testing.

GC withdrawal symptoms were less frequently reported, although more commonly reported by non-endocrinologists (1.7% and 10%, respectively). Other challenges to weaning GCs are summarised in figure 3B.

## Discussion

Our study highlights the heterogeneous approaches used to wean GCs, including differences in the GC doses at which the HPA axis would be evaluated and the rate at which GCs would be weaned. Nearly half of non-endocrine specialists would wean the GC dose slowly, with 10% referring to local endocrine services to help supervise this. Referral to endocrinology may not be helpful unless there is a history of adrenal crises, or for those who have prolonged HPA axis suppression GCs.^7 8^ It may be that lack of resources necessitates referral in lower-income countries in Southeast Asia.

Although it is difficult to fully compare region-specific practices, we observed common patterns. Switching to replacement-dose hydrocortisone with subsequent weaning was more frequent in SEA than in UK, whereas immediate long-term hydrocortisone (without planned wean) and reducing to 3 mg prednisolone before weaning were relatively more common in the UK. These observed regional differences likely reflect contextual factors, including access to Synacthen, availability of 1 mg prednisolone tablets and local care pathways that may encourage specialist referral in complex or resource-limited settings.

Among the endocrine specialists who responded, there was variation in the approach to investigating and managing a patient with a morning cortisol of 98 nmol/L while taking long-term 5 mg prednisolone. 35.4% of endocrinologists would wean the prednisolone dose below 5 mg before assessing endogenous cortisol recovery, compared with nearly 10% of respondents who would accept this result as confirmatory of adrenal insufficiency and continue 5 mg prednisolone indefinitely. Despite guidelines recommending not to switch prednisolone to hydrocortisone, this was the preferred GC for endocrinologists to use to initiate weaning. A recent retrospective study, however, demonstrated that conversion from prednisolone to hydrocortisone did not enhance HPA recovery in dose-matched individuals.^10^ The recovery rate in the prednisolone group (n=10) was 70% vs 15% in those who converted to hydrocortisone (n=13).^10^ However, such studies are limited by their retrospective design and robust prospective evidence comparing weaning on prednisolone and hydrocortisone is required.

The UK’s National Institute for Health and Care Excellence guidelines recommend a slow taper in people taking GCs for greater than 12 weeks and endorse the Imperial Centre for Endocrinology prednisolone withdrawal regimen.^8^ This is a 24-week weaning protocol that aims to allow recovery of endogenous cortisol production while minimising relapse of the underlying GC condition and GC withdrawal symptoms. However, there is wide variation in prednisolone metabolism, which may explain some of the difficulty in weaning.^11^

An SST can be used as a dynamic test during GC weaning to observe HPA axis recovery and was the choice in 42.2% of endocrine specialists. Interestingly, nearly one fifth of endocrine respondents reported that they would continue 5 mg prednisolone if there was evidence of adrenal suppression on an SST, despite 5 mg prednisolone being the cause of this.

However, the use of the SST is limited by potential timing and sampling issues and variability in interpretation.^12^ Differences in calibration, specificity of immunoassays and cortisol cut-off values may all impact the interpretation of the results and impact GC weaning.^13^ Although there is a low risk of hypersensitivity, it is time-consuming with significant resource implications.^14^ It is therefore important to ensure interpretation of the SST results is in conjunction with the patient’s symptoms during the weaning regimen. In complex scenarios where individuals are unable to tolerate GC weaning, specialist endocrinology input may be helpful in the event of a new diagnosis of primary adrenal insufficiency.

It is also not known why some patients are able to fully wean GCs compared with others who develop permanent HPA axis suppression and require long-term GC replacement. Of note, relapse of the underlying condition was most frequently reported barrier preventing successful weaning in both respondent groups. This suggests that weaning (and referral to endocrinology) should only be started once there is adequate underlying disease control (eg, from non-glucocorticoid based therapies), and the patient is taking a physiological prednisolone dose.^15^

GC withdrawal symptoms were more commonly reported by non-endocrine physicians, although distinguishing these from symptoms of adrenal insufficiency is challenging.^16^,^18^ There is therefore a need to ensure patients are counselled appropriately and for future studies to determine the most effective tapering strategies.

This study was limited by the two slightly different single case-based scenarios, which potentially limits the generalisability and significance of the results. Although the survey was anonymous, answers to the questions relied on self-reported data, which may be subject to recall bias. The respondent time frame of 3 months and design of the survey in English, which may have also limited participation. In addition, lack of availability of Synacthen and lower strength doses of prednisolone in some parts of the world may have affected the respondents’ choices and should be considered in future work. As of October 2025, 1 mg prednisolone is now on the WHO list of essential medicines.

However, to our knowledge, this is the largest multinational survey to date assessing current practices and barriers associated with GC weaning across a large and diverse group of endocrinologists and non-endocrine specialists in both the UK and in Southeast Asia.

Our findings support the urgent need for prospective randomised controlled trials to evaluate and compare weaning protocols.

## Conclusions

Despite the two slightly different clinical scenarios, there is variation in the way GCs are weaned both among endocrine and non-endocrine specialists. Although recent guidelines provide a framework for investigating and managing patients no longer requiring therapeutic GC doses, this study demonstrates relapse of the underlying condition during weaning is common, and endocrinology input may not be appropriate when this occurs. There remains a need to develop an evidence-based approach for safe and effective GC weaning and HPA axis assessment, to avoid the health burden associated with long-term GC therapy.

## Supplementary material

10.1136/bmjopen-2025-107269 online supplemental file 1

10.1136/bmjopen-2025-107269 online supplemental file 2

## Data Availability

All data relevant to the study are included in the article or uploaded as supplementary information.
