# Supplementary material for: Weaning patients off long-term prednisolone: a survey of physicians’ practice in the UK and Southeast Asia
Source: BMJ Open. 2025 Dec 30;15(12):e107269. doi: 10.1136/bmjopen-2025-107269 (PMC12766809; doi:10.1136/bmjopen-2025-107269)
Supplement: online supplemental file 2 [file bmjopen-15-12-s002.docx]

**Supplementary Materials**

**Questionnaire sent to endocrine respondents.**

| 1. Have you ever treated patients with steroid-induced adrenal insufficiency  - Yes - No  1. A 45-year-old patient with asthma has a recently been started on immunologic therapy.  They have a **15-year history of taking long-term once daily 5mg prednisolone**, which the respiratory team would now like to discontinue.   A **0900 am cortisol before prednisolone is 98 nmol/L** (last dose 24 hours ago). They are otherwise well. Which of the following investigations would you undertake in your normal clinical practice? In all cases, tests are done before any steroids are taken. There are no wrong answers.  Please answer as you would do in real life. If you choose others, please kindly specify   - Short Synacthen test (including baseline ACTH) - Insulin tolerance test - Glucagon stress test - Metyrapone suppression test - Early morning salivary cortisone - No further investigations while the patient is on this dose of steroid - No further investigations at all - Others   3. How would you normally manage the patient?  There are no wrong answers.  Please answer as you would do in real life.   - Switch to replacement dose hydrocortisone and continue this long-term - Switch to replacement dose hydrocortisone and slowly wean the patient off hydrocortisone completely. - Continue 5mg prednisolone long-term - Reduce prednisolone to 3mg once daily and continue this long-term - Reduce to prednisolone to 3mg once daily and slowly wean the patient off prednisolone completely. - Others  1. Which of these investigations would you repeat, after an interval, if any?  - 0900 am cortisol - Short synacthen test (SST) - Insulin tolerance test (ITT) - Glucagon stress test (GST) - Metyrapone suppression Test - Early morning salivary cortisone - No investigations at all - Other (please specify)  1. For the above scenario, would you routinely do any other investigations to optimise patients’ management (e.g. hydrocortisone day curves or prednisolone levels?). If you choose others, please kindly specify  - Yes - No   6. How often would you routinely follow up this patient in the Endocrine clinic?   - Every 3 months - Every 6 months - Every 12 months - None   7. Would this follow-up be face to face or remote follow-up?   - Face-to-face appointment - Remote follow-up (telephone, video or email) - Combination of face-to-face and remote follow-up - Not applicable   8. Would you consider discharging this patient from Endocrine follow-up? If others, please specify   - No - Yes - Others   9. Which of the following do you find is the commonest cause of failure to wean glucocorticoids in patients referred by other specialties?   - Relapse of the patient’s underlying condition requiring treatment doses of steroids - Symptoms of adrenal insufficiency - Flat SST results - Other (please specify)   10. Which specialties refer patients to you for steroid weaning? (Tick all that apply)   - Rheumatology - Respiratory - Dermatology - Gastroenterology - Neurology - Oncology - Other (please specify)   11. Do you have a local protocol or pathway for steroid weaning so that non-endocrinologists can safely wean patients from steroids without the need to refer to endocrinology?  12. Where do you work?   - Secondary care Hospital - Community hospital - Tertiary Centre - Primary Care   13. What is your job role?   - Endocrine Specialist Nurse - Senior House Officer/Medical Officer - Endocrine Specialist Registrar/Resident - Consultant - General Practitioner - Other   14. What other challenges would you like to highlight in the practice of steroid weaning in your patients? |
| --- |

**Questionnaire sent to non-endocrine respondents**

| 1. Do you treat patients with long-term glucocorticoid steroids?  - Yes - No   2. You have started a 45-year-old patient on **once daily 10mg prednisolone**, which you plan to discontinue, as the condition the prednisolone has been used to treat is now in remission. Which of the following do you do in your normal clinical practice? Tick as many as apply. There are no wrong answers.  Please answer as you would do in real life.   - Stop prednisolone without weaning down the dose. - Wean the prednisolone slowly (e.g. 1mg/month) - Wean the prednisolone over 2 weeks (reduce by 1mg daily) - Refer to Endocrinology to supervise weaning - Check a random cortisol value - Check an early morning cortisol (prior to prednisolone dose) - Arrange a short Synacthen test for the patient - Other (please specify)   3. Which of the following do you find is the commonest cause of failure to wean glucocorticoids in patients?   - Relapse of the patient’s underlying condition requiring treatment doses of steroids - Symptoms of adrenal insufficiency - Failed short Synacthen test - Symptoms of glucocorticoid withdrawal - Other (please specify)   4. Which speciality are you from? (Tick all that apply)   - Rheumatology - Respiratory - Dermatology - Gastroenterology - Neurology - Oncology - Haematology - Nephrology   5. Do you work at a District General Hospital (DGH) or Tertiary Centre?   - District General Hospital - Tertiary Centre   6. What is your job role?   - Specialist Nurse - Junior Doctor (FY1 to IMT3)/House Officer-Senior House Officer - Specialist Registrar (ST4 or above)/Senior Doctor - Consultant - Other |
| --- |
