# Supplementary material for: Weaning patients off long-term prednisolone: a survey of physicians’ practice in the UK and Southeast Asia
Source: BMJ Open. 2025 Dec 30;15(12):e107269. doi: 10.1136/bmjopen-2025-107269 (PMC12766809; doi:10.1136/bmjopen-2025-107269)
Supplement: Supplementary file 3 [file bmjopen-2025-107269.reviewer_comments.pdf]

PEER REVIEW HISTORY

BMJ Open publishes all reviews undertaken for accepted manuscripts. Reviewers are asked to complete a checklist review form and are provided with free text boxes to elaborate on their assessment. These free text comments are reproduced below.

ARTICLE DETAILS

Title (Provisional)

Weaning patients off long-term prednisolone: A survey of physicians’ practice in the UK and Southeast Asia

Authors

Lazarus, Katharine; Eng, Pei Chia; narula, kavita; Sharma, Angelica; Choudhury, Sirazum; Papadopoulou, Deborah; Martin, Niamh M; Wernig, Florian; Tan, Tricia; Meeran, Karim

---

VERSION 1 - REVIEW

**Reviewer**	**1**
**Name**	**Bizzarri, Carla**
**Affiliation**	**Bambino Gesù Children Hospital (IRCCS)**
**Date**	**07-Aug-2025**
**COI**	**None**

The manuscript is well-written but does not present any significantly innovative findings regarding weaning patients off long-term glucocorticoid therapy.

The authors conducted an online survey targeting a group of endocrine responders and a group of non-endocrine specialists to assess how glucocorticoids are weaned in patients undergoing long-term treatment. However, it is unclear why the survey focused exclusively on prednisolone therapy, given that numerous synthetic steroids are used as anti-inflammatory and immunosuppressive agents.

These steroids vary in potency and duration of action, which affects their potential to suppress the hypothalamic-pituitary axis; consequently, weaning protocols may differ substantially. Additionally, the approach to weaning may vary depending on the primary disease, its severity, duration of therapy and the frequency of relapses.

The use of a single case-based scenario to evaluate how participants would manage glucocorticoid weaning clearly limits the generalizability and significance of the results.

mino comment

Table 1: there is mistake in the total number of respondents (Line tertiary centre)

**Reviewer**	**2**
**Name**	**Hee, Nicholas Ken Yoong**
**Affiliation**	**Universiti Malaya, Medicine**
**Date**	**09-Sep-2025**
**COI**	**None**

This is an important paper that aims to explore practices of endocrinologists and non endocrinologists with regard to glucocorticoid weaning practices, in view of recently published recommendations on the same topic.

Issues are highlighted as below:

1. 2 separate versions of questionnaires were given to endocrinologists and non endocrinologists with 2 different clinical scenarios. While this may have been done to give a simpler case to non endocrinologists, the authors state at the end of their article in line 290-291 that there is a "huge variation in the way GCs are weaned amongst both endocrine and non endocrine specialists". This may not be a fair conclusion as they were not led in with the same scenario, and hence may arrive at a different conclusion.

2. There is significant heterogeneity in respondents, with many more endocrine consultants responding in the UK, but less in SouthEast Asia. Places of practice were also included in the demographics or respondents, but authors have not drawn any conclusion as to whether this has affected practice. This may affect availability of SST and/or lower doses of glucocorticoids.

3. In line 177 - 184, authors attempt to describe the different practices of endocrinologists, referencing Figure 2B. This was not easy to follow as the numbers on Figure 2B do not match the figures in the text at all. For example, it is stated that 59.6% would switch from prednisolone to hydrocortisone, but the number in the figure is 56.6%, and for SouthEast Asia only but not UK. Perhaps this is because the proportion of UK/SEA is not the same, but the authors should make this clear.

4. In line 228-231, authors highlight that many non endocrinologists still refer to endocrinologists to wean GCs despite guidance not to refer unless certain scenarios are met. It is not made clear why the referral happens in the discussion. Perhaps a lack of resources, or lack of lower dose prednisolone/availability of SST necessitates referral in lower income countries in South East Asia. This should not be automatically perceived as lack of compliance to guidelines, especially as authors state 69.2% of endocrinologists do use SST regularly.

5. In lines 238-240 and 255-258, authors highlight the lack of compliance to guidelines that do not require a switch to hydrocortisone. However, it is already stated in the limitations by

respondents in Figure 3B that one of the reasons for this is the lack of availability of 1mg prednisolone. This should be made apparent in the discussion as well.

6. As one of the strengths of the study is its multinational respondents, it is important that authors address the heterogeneity of respondents in terms of countries of practice, and describe if this has led to a difference in prescribing and referral practices. This was not done.

7. In line 182 and 193, authors cite respondents answering "life-long GC replacement". However in the questionnaire, the answer that respondents are given is "Switch to replacement dose hydrocortisone and continue this long-term" and "Reduce prednisolone to 3mg once daily and continue this long-term". It is debatable as to whether respondents understand "long term" to mean "life-long". "Long term" may mean 6 months or 1 year before reassessment, depending on interpretation. Concluding that physicians equate "long term" to "life-long" is erroneous, considering that is not what was asked in the question.

VERSION 1 - AUTHOR RESPONSE

Reviewer: 1
Dr. Carla Bizzarri, Bambino Gesù Children Hospital (IRCCS)
Comments to the Author:
The manuscript is well-written but does not present any significantly innovative findings regarding weaning patients off long-term glucocorticoid therapy.
The authors conducted an online survey targeting a group of endocrine responders and a group of non-endocrine specialists to assess how glucocorticoids are weaned in patients undergoing long-term treatment. However, it is unclear why the survey focused exclusively on prednisolone therapy, given that numerous synthetic steroids are used as anti-inflammatory and immunosuppressive agents.

These steroids vary in potency and duration of action, which affects their potential to suppress the hypothalamic-pituitary axis; consequently, weaning protocols may differ substantially. Additionally, the approach to weaning may vary depending on the primary disease, its severity, duration of therapy and the frequency of relapses.

The use of a single case-based scenario to evaluate how participants would manage glucocorticoid weaning clearly limits the generalizability and significance of the results.

Authors' response:
We thank the reviewer for this, and we agree that weaning protocols and physicians' approaches may vary and have updated the methods section to highlight this (lines 133-135).

We focused on prednisolone because it is widely used for chronic indications in many regions and, in many services, patients on longer-acting agents are routinely switched to prednisolone before tapering. This provided a common comparator scenario across

specialties and settings, and we have updated the methods section to clarify this (lines 140-143).

We agree that the use of a single case scenario would limit generalizability and significance of the results and have updated the discussion section to clarify this (lines 292-293).

minor comment
Table 1: there is mistake in the total number of respondents (Line tertiary centre)
Authors' response: We thank the reviewer for highlighting this and we apologise this was missed. We have updated the table with the corrected total number of participants (Table 1, corrected number 118).

Reviewer: 2
Dr. Nicholas Ken Yoong Hee, Universiti Malaya
Comments to the Author:
This is an important paper that aims to explore practices of endocrinologists and non endocrinologists with regard to glucocorticoid weaning practices, in view of recently published recommendations on the same topic.

Issues are highlighted as below:

1. 2 separate versions of questionnaires were given to endocrinologists and non endocrinologists with 2 different clinical scenarios. While this may have been done to give a simpler case to non endocrinologists, the authors state at the end of their article in line 290-291 that there is a "huge variation in the way GCs are weaned amongst both endocrine and non endocrine specialists". This may not be a fair conclusion as they were not led in with the same scenario, and hence may arrive at a different conclusion.

Authors' response:
We thank the reviewer for this and agree that this could be misinterpreted. We have updated the conclusion to clarify that the scenarios were different (line 307).

2. There is significant heterogeneity in respondents, with many more endocrine consultants responding in the UK, but less in Southeast Asia. Places of practice were also included in the demographics or respondents, but authors have not drawn any conclusion as to whether this has affected practice. This may affect availability of SST and/or lower doses of glucocorticoids.

Authors' response: We thank the reviewer for highlighting this and we agree that observed differences may partially reflect respondent composition and regional resource availability (e.g., SST access, availability of low-dose formulations). We acknowledge that our findings should be interpreted as descriptive rather than

population-representative. We have updated the discussion section to add this as a limitation of the study (lines 296-298).

3. In line 177 - 184, authors attempt to describe the different practices of endocrinologists, referencing Figure 2B. This was not easy to follow as the numbers on Figure 2B do not match the figures in the text at all. For example, it is stated that 59.6% would switch from prednisolone to hydrocortisone, but the number in the figure is 56.6%, and for SouthEast Asia only but not UK. Perhaps this is because the proportion of UK/SEA is not the same, but the authors should make this clear.

Authors' response: We thank the reviewer for highlighting this and apologise for the confusion. The apparent mismatch arose because Figure 2B reports *region-specific* percentages using region-specific denominators (UK n = 162; Singapore/SEA n = 76), whereas in the manuscript, the results paragraph used pooled cohort percentages (Total N = 238). We have updated Figure 2B and the manuscript to highlight the total number of responses from each region and the total responses overall (lines 182-188):

Figure 2B compares the proportion of respondents in the different regions, demonstrating the wide variability in GC weaning practices. The most common approach was to switch to replacement-dose hydrocortisone (50.4%) and then slowly wean off completely, compared to 27.7% who opted to wean on prednisolone. A small proportion (4.6%) said they would continue long-term 5mg prednisolone compared with 3.4% who would reduce the prednisolone dose to 3 mg first and then continue this long-term.

4. In line 228-231, authors highlight that many non-endocrinologists still refer to endocrinologists to wean GCs despite guidance not to refer unless certain scenarios are met. It is not made clear why the referral happens in the discussion. Perhaps a lack of resources, or lack of lower dose prednisolone/availability of SST necessitates referral in lower income countries in South East Asia. This should not be automatically perceived as lack of compliance to guidelines, especially as authors state 69.2% of endocrinologists do use SST regularly.

Authors' responses:
We agree that referral by non-endocrinologists may be appropriate and are likely to be resource-driven. We also agree that in some settings, limitation of low-dose prednisolone and tablet splitting constraints may influence the preference of physicians to switch to prednisolone during weaning. We acknowledge that this observed preference may be more of a pragmatic dose-titration strategy rather than a deviation from guidance. Thus, we have added in the discussion (lines 296-298) the plausible drivers that may drive referrals (e.g. limited access to SST, lack of low dose formulation for prednisolone, the need for dynamic testing) and reframed the interpretation away from "non-compliance."

5. In lines 238-240 and 255-258, authors highlight the lack of compliance to guidelines that do not require a switch to hydrocortisone. However, it is already stated in the

limitations by respondents in Figure 3B that one of the reasons for this is the lack of availability of 1mg prednisolone. This should be made apparent in the discussion as well.

Authors' response: We thank the reviewer for this comment, and we have now described regional preference in tapering approach and presented it descriptively with appropriate denominators (Updated figure 2B). We have also added the lack of low-dose prednisolone or SST as an apparent limitation in our discussion section (lines 296-298).

It is also worth noting, that as of October 2025, 1 mg prednisolone is now on the WHO list of essential medicines.

6. As one of the strengths of the study is its multinational respondents, it is important that authors address the heterogeneity of respondents in terms of countries of practice and describe if this has led to a difference in prescribing and referral practices. This was not done.

Authors' response:
We thank the reviewer for highlighting this and we agree that respondent heterogeneity by region may influence prescribing and referral practices. We have now updated this by presenting the break down of weaning practices using region-specific denominators with pooled total values shown to provide context (Updated Figure 2B). Given the sample size imbalance and the study's descriptive design, it is difficult to fully compare region-specific practices. We have also updated the discussion section (lines 237-243) to further detail this.

7. In line 182 and 193, authors cite respondents answering "life-long GC replacement". However in the questionnaire, the answer that respondents are given is "Switch to replacement dose hydrocortisone and continue this long-term" and "Reduce prednisolone to 3mg once daily and continue this long-term". It is debatable as to whether respondents understand "long term" to mean "life-long". "Long term" may mean 6 months or 1 year before reassessment, depending on interpretation. Concluding that physicians equate "long term" to "life-long" is erroneous, considering that is not what was asked in the question.

Authors' response:
We thank the reviewer for highlighting this and apologise for this oversight. We have updated the manuscript to correct this (lines 49 and 196).

Reviewer: 1

If you have selected ‘Yes’ above, please provide details of any competing interests.: Not applicable

Reviewer: 2
If you have selected ‘Yes’ above, please provide details of any competing interests.: Not applicable

VERSION 2 - REVIEW

**Reviewer**	**2**
**Name**	**Hee, Nicholas Ken Yoong**
**Affiliation**	**Universiti Malaya, Medicine**
**Date**	**29-Oct-2025**
**COI**	

Thank you for addressing my comments. I believe this is an important research topic to address, especially in a region like SouthEast Asia where glucocorticoid use is prevalent, but data is often scarce. I have no further requests for revision at this time.

VERSION 2 - AUTHOR RESPONSE

Reviewer: 2
Dr. Nicholas Ken Yoong Hee, Universiti Malaya
Comments to the Author:
Thank you for addressing my comments. I believe this is an important research topic to address, especially in a region like SouthEast Asia where glucocorticoid use is prevalent, but data is often scarce. I have no further requests for revision at this time.

We thank the reviewer for these comments, and agree, this is an important area that requires future work to address weaning strategies to mitigate glucocorticoid-related morbidity.

Reviewer: 2
If you have selected ‘Yes’ above, please provide details of any competing interests.: Not applicable
